# Determinants of intention with remote health management service among urban older adults: A Unified Theory of Acceptance and Use of Technology perspective

**DOI:** 10.3389/fpubh.2023.1117518

**Published:** 2023-01-26

**Authors:** Wenjia Li, Jingjing Gui, Xin Luo, Jidong Yang, Ting Zhang, Qinghe Tang

**Affiliations:** ^1^College of Communication and Art Design, University of Shanghai for Science and Technology, Shanghai, China; ^2^School of Creativity and Art, Shanghai Tech University, Shanghai, China; ^3^School of Design and Art, Shanghai Dianji University, Shanghai, China; ^4^Shanghai East Hospital, Tongji University, Shanghai, China

**Keywords:** remote health management, behavioral intention, UTAUT, structural equation model, user experience, aging design

## Abstract

**Background:**

Although older adults health management systems have been shown to have a significant impact on health levels, there remains the problem of low use rate, frequency of use, and acceptance by the older adults. This study aims to explore the significant factors which serve as determinants of behavioral intention to use the technology, which in turn promotes actual use.

**Methods:**

This study took a total of 402 urban older adults over 60 years to explore the impact of the use behavior toward remote health management (RHM) through an online questionnaire. Based on the Unified Theory of Acceptance and Use of Technology (UTAUT), the author adds four dimensions: perceived risk, perceived value, perceived interactivity and individual innovation, constructed an extended structural equation model of acceptance and use of technology, and analyzed the variable path relationship.

**Results:**

In this study, the factor loading is between 0.61 and 0.98; the overall Cronbach's Alpha coefficients are >0.7; The composite reliability ranges from 0.59 to 0.91; the average variance extraction ranges from 0.51 to 0.85, which shows the good reliability, validity, and discriminant validity of the constructed model. The influencing factors of the behavioral intention of the older adults to accept the health management system are: effort expectation, social influences, perceived value, performance expectation, perceived interactivity and perceived risk. Effort expectation has a significant positive impact on performance expectation. Individual innovation positively impacts performance expectation and perceived interactivity. Perceived interactivity and behavioral intention have a significant positive effect on the use behavior of the older adults, while the facilitating conditions have little effect on the use behavior.

**Conclusions:**

This paper constructs and verifies the extended model based on UTAUT, fully explores the potential factors affecting the use intention of the older adult users. According to the research findings, some suggestions are proposed from the aspects of effort expectation, performance expectation, perceived interaction and perceived value to improve the use intention and user experience of Internet-based health management services in older adults.

## 1. Introduction

Aging has become one of the major problems faced by all countries in the world. In China, with the increase in the proportion and number of the older adult population, the serious aging, the coexistence of multiple chronic diseases, and the increase in medical expenses, this has brought a heavy burden of older adult care and huge economic challenges to medical institutions, older adults care institutions and even society. The health of the older adults has risen to a national strategic problem, and it is urgent to explore and open up new ways to manage the health of the older adults.

The concept of “health management” was first born in the United States and Canada. It specifically refers to the monitoring of health status, analysis of health data, evaluation and prediction of different populations, proposal of corresponding health plans, intervention and management of various potential risk factors, and supply of professional health guidance and services (1). The management system for the older adults derived from telemedicine (2) is based on Internet of Things, Internet of Systems and wireless body area networks, providing the older adults with an online self-health management platform to realize health information collection, health assessment, health consultation, emergency medical assistance and other functions. It has the advantages of real-time monitoring, two-way data transmission, online communication (3). Although older adults health management systems have been shown to have a significant physical and psychological impact on health levels (4) there remains the problem of low use rate, frequency of use, and acceptance of health management systems by the older adults. Therefore, it is meaningful to construct a health management system based on telemedicine for the older adults at present.

This study will focus on the potential older individuals who can actively use telemedicine, eliminate the special populations with senile dementia, trauma and tumors, and integrate previous studies to divide the health management content into four categories: health monitoring, chronic disease management, rehabilitation management, and accidental injury prevention. The Unified Theory of Acceptance and Use of Technology (UTAUT) is widely used to analyze users' usage behavior and intention. We will make appropriate expansion of the model and analyze the influencing factors of use behavior in older individuals through empirical research. The theoretical background and research hypotheses will be introduced in detail below, the results of the data and the final model will be analyzed, and the findings, significance, limitations, and future prospects of this study will be discussed.

## 2. Literature overview

### 2.1. Evolution of health management model and corresponding theoretical research

Health management is a systematic process of comprehensively monitoring, analyzing, evaluating, providing targeted scientific health information and intervening on potential health risk factors for the health of individuals or groups, and planning, organizing, directing, coordinating and controlling information and resources related to human health (5). Health management of the older adults is a relatively new concept derived from the concept of health management. The older individuals are characterized by susceptibility to disease and a high proportion of chronic diseases, and is the key population for health management. Health management of the older adults refers to the comprehensive detection, analysis and evaluation of the health status of the older individuals and groups, and then provide health counseling and guidance to the older adults, develop health risk factor intervention plans for the older adults and carry out the whole process of chronic disease prevention and control, disease diagnosis and treatment, rehabilitation nursing, long-term care, etc. for the older adults.

At present, there have been some studies on the health management of the older adults. Under the multiple burdens of aging and compression of health care resources, the United States government has shifted the focus of health management from “diagnosis and treatment” to “prevention and maintenance.” A national health management program, the “Health People1990” program, was developed (6), which set the older adults as a national goal. The United Kingdom and other European countries have followed the relevant approach. The United Kingdom has established a variety of projects such as the National Health Service System and NSF for older people (7). Japan is a country with serious aging and early health management in Asia. The success of “u-Japan Strategy” has brought about the development of telemedicine in Japan (8).

In 1998, China introduced the concept of “health management” and based on the advanced experience of health management in the United States, and initially established a primary health management system from health examination, health file management, health risk assessment, health education, healthy lifestyle guidance, intervention of health risk factors, disease management. At present, most of the representative health management systems rely on Internet, cloud platform and other information and communication technologies, develop and integrate internal and external information resources, deepen the form and degree of cooperation with medical service providers through mobile application (WeChat, etc.) SMS, telephone and other means, and establish a core system for health management business. Nowadays, with the development of science and technology and the mature subdivision of health management, the Internet of Things, 5G network, cloud computing platform, wearable and implantable sensing technology are bringing a brand-new medical ecosystem that can rely on each other to China.

Although the health management system has become a link in the daily life of Chinese people, the search of the relevant literature (9, 10) revealed that there are significant deviations in its acceptance, which are susceptible to various factors such as age, education, income and self-perceived health status. Studies have shown that the applicability of telemedicine to the older adults has not become an obstacle to their acceptance of telemedicine services, and the older adults are very interested in the use of telemedicine services (11). There are many studies on the factors affecting the health acceptance of the older adults abroad, while the Chinese studies are not rich enough in this area. The content of this paper is the acceptance of the new health management model under the integration of telemedicine and the factors that affect the behavioral intention of the older adults to use the remote health management system.

### 2.2. Definition of telemedicine and its application in older health management

The World Health Organization defines telemedicine as “health care practice using interactive audiovisual and data communication.” This includes the supply of health care services, diagnosis, counseling, treatment, and health education and transfer of medical data (12). Telemedicine is divided into three types: synchronous and asynchronous, data transmission and storage, automatic and robotic telemedicine services (13). As an open, shared and evolving science, telemedicine continuously integrates new advances in information and communication technologies to meet socio-economic development and changing public health needs to achieve the key objectives of providing medical services and information exchange across regions and without time constraints (14).

With the development of various emerging technologies, as well as more powerful and convenient handheld devices (15), telemedicine has achieved the use of intelligent devices to record and upload health data at any time, and conduct information consultation with doctors anytime and anywhere (16). The transformation of telemedicine mode has changed health management from medical institution-led to “patient-centered” (17) individual spontaneous management. In this evolution trend, health management has been smoothly integrated into telemedicine, thus there is a saying of remote health management. Remote health management refers to a new health management model that combines Internet, Internet of Things, cloud computing and other technologies with monitoring equipment, transmits the collected health data to the monitoring platform, analyzes and evaluates the health data of patients through the monitoring platform, and gives individualized reminders, suggestions and interventions according to the health data of patients (18).

According to relevant statistics from the China National Committee on Aging, 75.8% of the older adults suffer from at least one chronic disease (19). This self-health management of chronic diseases in the older adults based on Internet of Things is to extend the coverage of medical services to families, emphasize the degree of individual independent participation on the basis of traditional health management, and shift the focus of monitoring, evaluation, intervention and tracking from medical institutions to families and individuals. With the help of Internet of Things, telemedicine and self-service medical model, older patients with chronic diseases may dynamically collect and analyze physiological signs information through physiological index monitors and health scales, monitor physical conditions in real time, and improve their self-health (20).

Remote rehabilitation is considered to be a means to provide rehabilitation services to patients at home, and its benefits lie in the ability to overcome geographical, physical, and cognitive impairments and compensate for the shortcomings of traditional treatments subject to hospitals (21). Such as remote rehabilitation using wearable muscle sensors and Kinect (22). Accidental injury, unlike common diseases, is physical injuries outside of everyday situations, and the prevention aims to provide personalized assistance services for the older adults in emergency situations (23). Falls tend to be the main cause of fatal and non-fatal injuries in the older adults (24), and remote fall monitoring allows the older adults to receive timely assistance (25). Remote monitoring can also be used to predict sudden hypertension in the older adults under special circumstances (26, 27). And instructional alerts are immediately sent to physicians in case of emergencies (28). Therefore, based on the above analysis, this paper defines telemedicine management in the older adults as health surveillance, chronic disease management, rehabilitation management, and accidental injury prevention.

### 2.3. Model analysis

The UTAUT theory model which was first proposed by Venkatesh et al. (29) is used in this study. It is a comprehensive model containing eight models and prominent theoretical models, which aims to predict and explain users' behavioral intention to use information technology and their use behavior. The model is composed of four core variables and four moderation variables. The four core variables are performance expectation, effort expectation, social influences and facilitating conditions, and the moderation variables include gender, age, experience, and voluntariness of use, of which performance expectation, effort expectation and social influences directly affect the behavioral intention, and the facilitating circumstances and behavioral intention directly affect the use behavior, and gender, age, voluntariness of use and experience play a moderating role (29). According to research, more than 40% of information technology failures may be due to lack of full recognition of how individuals or organizations accept and use technology, and information technology can better provide services only if it is accepted and used by users (30). The popularization and application of the health management system is not only related to technology and services, but also closely related to the acceptance of the older adults. At present, the use frequency of emerging health management systems by the older adults is not high, and the acceptance of emerging health management systems is far lower than that of other age groups. Therefore, this paper will use the UTAUT model combined with the characteristics of the older adults and health management system to study the factors affecting the behavioral intention of the older adults to use the health management system.

## 3. Hypothesis and research model

### 3.1. Model presentation

Acceptance refers to the behavioral intention within the user group to use ICT to accomplish tasks supported by their design. Among them, the Unified Theory of Acceptance and Use of Technology (UTAUT) model is a derived model of the Technology Acceptance Model (TAM) model, which is able to explain 69% of intention technology acceptance, while other models can explain only about 40% of technology acceptance (29). Many scholars have studied technology acceptance using the UTAUT model. Some scholars (31, 32) used modified UTAUT models to analyze health care professionals' acceptance of electronic medical records. Other scholars (33, 34) explored possible influencing factors of mobile medicine using the UTAUT model. Therefore, UTAUT is considered to be the most consistent model mentioned above, and it is also the most active model used in technology acceptance research in the field of healthcare (35). The application of UTAUT model to the acceptance and use of information and communication technologies (ICT) by the older adults within the healthcare domain will expand the understanding of the robustness of the model in explaining the acceptance and use.

**Figure 1:**
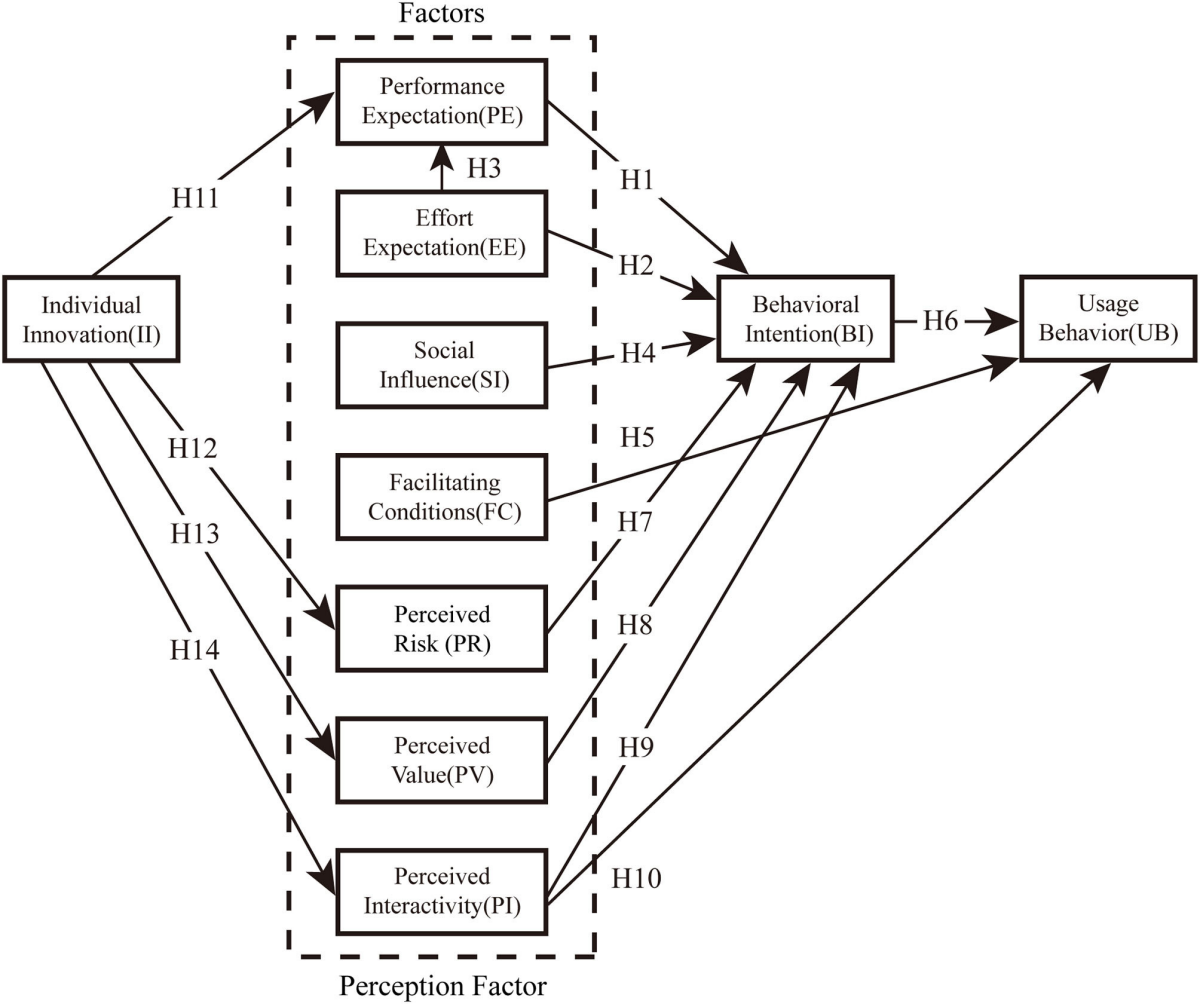
The proposed research model.

To adapt to the research presented here, we will adjust these structures and definitions from the original UTAUT model. The UTAUT model was originally used to measure the acceptance of technology in the organizational environment. When adjusting the measurement factors of the model, the perspective of the older adults and the background of health care must be considered (36). In this paper, we retain the four core elements in the original UTAUT model: Performance Expectancy (PE), Effort Expectancy (EE), Social Influence (SI), and Facilitating Conditions (FC). The reasons for retaining the core variables are as follows: these four core elements are important indicators of studying users' behavioral intention to use information technology. The original author concluded after validating the UTAUT model in six different organizations that performance expectation, effort expectation, and social influence are direct determinants of behavioral intention, and behavioral intention and facilitating conditions directly affect use behavior, so they are retained (29). The four moderation variables (gender, age, voluntariness of use and experience) are removed from the original model. The reasons for removing the moderation variables are as follows. The subject of this study is the older individuals over 60 years old, whose age is relatively concentrated, so the moderation variable of age is removed; secondly, the development of the older health management system in China is still not very mature, and there is less reference in practical experience and voluntariness of use in terms of acceptance, so the experience and voluntariness of use are not included. In order to more accurately and comprehensively explore the influencing factors of the older adults accepting health management system, four variables of perceived interactivity, perceived value, perceived risk and personal innovation are added according to the characteristics of the older adults and health management system to jointly construct the initial model framework suitable for this study. In order to verify the relationship between performance expectation, effort expectation, social influences, facilitating conditions, perceived risk, perceived value, perceived interactivity, personal innovation and their behavioral intention to use remote health management, we developed a conceptual model based on the UTAUT model outlined in the literature review (Model Figure 1), defining the relevant variables of the model and proposing research hypotheses.

### 3.2. Formulation of the research hypotheses

#### 3.2.1. Performance expectancy

Performance expectancy refers to the degree to which an individual believes that using the system will help him or her to attain gains in job performance. Performance expectation comes from the perceived usefulness introduced in the original TAM model (29, 37). In the context of telemedicine, usefulness can be regarded as the degree to which the older adults subjectively believe that the use of remote health management systems can improve or help their own health benefits and reduce personal health threats. Of the 116 studies on the relationship between performance expectation and behavioral intention, 93 studies (80%) found a significant relationship between the two (38). The key for users to accept information technology is to perceive the usefulness of technology. The fear that technology does not achieve the expected performance usually leads to the behavior of older individuals unwilling to use the technology (39). Many studies in the medical field have proved that performance expectations have a positive impact on users' behavioral intention (40). When the older adults perceive that the health management system can improve the efficiency of their own health management, which is beneficial to themselves, the behavioral intention is strong. Therefore, the hypothesis is put forward:

H1: Performance expectation has a positive impact on the behavioral intention of the older adults to use remote health management.

#### 3.2.2. Effort expectancy

Effort expectation refers to the degree of ease associated with the use of the system and come from perceived ease of use introduced in the TAM model (29). In this paper, it refers to the degree of ease that the older adults perceive to use the health management system. Previous studies have shown that effort expectation has a strong impact on the extent to which users accept new technologies, especially for the older individuals (41), who tend to encounter more barriers to use new technologies than other ages, and thus tend to have a lower behavioral intention to use new technologies (42). Mohammed-Issa demonstrated that perceived ease of use had a positive effect on perceived usefulness (43). That is the ease of use of telemedicine services by the older individuals strongly affects their behavioral intention to use the service and affects their acceptance behavior (36). If the health management system is simple and easy to operate, the older adults are willing to use it and will be easier to find the usefulness of the management system. Therefore, the hypothesis is put forward:

H2: Effort expectation has a positive impact on the behavioral intention of the older adults to use remote health management.
H3: The effort expectation of the older adults for remote health management positively affects the performance expectation.

#### 3.2.3. Social influence

Social influence refers to the degree to which an individual perceives that important others believe he or she should use the new system (29). It here refers to the extent to which the older adults use health management systems because they are influenced by people around them. Of the 115 studies investigating the relationship between social influence and behavioral intention, 86 studies (75%) found that social influence had a significant impact on behavioral intention (38). In the field of medical health, Lu et al. found that social influence had a positive impact on users' behavioral intention to use information technology (44). Then, in the social environment where the older adults live, their subjective views on new technologies often depend on the evaluation of new technologies by their relatives and friends or children and grandchildren. When they feel support, encouragement or recommendation, the behavioral intention of older adults to use it will be relatively higher, and the older adults will be more willing to try it. Therefore, the hypothesis is put forward:

H4: Social influence has a positive impact on the behavioral intention of the older adults to use remote health management.

#### 3.2.4. Facilitating conditions

Facilitating conditions refer to the degree to which an individual believes that an organizational and technical infrastructure exists to support use of the system (29). In this paper, it refers to the extent to which the older adults have conditions that can be supported when using remote health management systems. Williams et al. investigated 54 studies of the relationship between facilitating conditions and behavioral intention and stated that 36 studies (67%) demonstrated a significant impact of facilitating conditions on use behavior (38). Yi et al. demonstrated in their study that facilitating conditions are direct determinants of use behavior of technology (45). The older health management system in this study is a remote online service platform that needs to be used on devices that support networking, and having devices that support the use of health management systems will promote the use of this management system by the older adults. Therefore, the hypothesis is put forward:

H5: Facilitating conditions have a positive impact on the behavior of the older adults to use remote health management.

#### 3.2.5. Behavioral intention

Behavioral intention refers to an indication of an individual's readiness to perform a given behavior. In this paper, it refers to the tendency of the older adults to use the remote health management systems. A review of the UTAUT model shows that of the 61 studies on the relationship between behavioral intention and use behavior, 51 (82%) studies proved that behavioral intention has a positive effect on use behavior (38). Venkatesh et al. (29) empirically demonstrated the significant positive effect of behavioral intention on the use behavior and suggested that behavioral intention is a valid influencing factor of actual use behavior. Therefore, the hypothesis is put forward:

H6: Behavioral intention of the older adults to use health management systems positively impacts their use behavior.

#### 3.2.6. Perceived risk

The original concept of perceived risk comes from psychology. Perceived risk can be understood as uncertainty about the possible adverse consequences of using a product or service. Uncertainty refers to the user's perception of whether a risk will arise when performing a certain behavior, that is, the risk to be taken before the decision is made; adverse consequences mainly refer to the dangerous consequences resulting from the occurrence of a risk (46). Considering the conservative psychological characteristics of the older adults and the relatively new characteristics of telemedicine, this paper defines the perceived risk as various risks that the older adults may subjectively judge before using telemedicine management, such as money risk, time risk, privacy risk, etc. Wu J et al. pointed out that perceived risk had a significant negative impact on behavioral intention of users, and users would hesitate or doubt due to problems such as asymmetry of information, diversification of information, and redundancy, which played a certain role in hindering the use of new things (47). As an emerging remote service platform, the older adults have various concerns due to technical barriers, which will reduce the behavioral intention to use remote health management systems. Therefore, the relevant hypothesis is put forward:

H7: Perceived risk has a negative impact on the behavioral intention of the older adults to use remote health management.

#### 3.2.7. Perceived value

From the perspective of consumer psychology, the perceived value of consumers was an evaluation of the overall consumption experience of the utility of products or services by consumers by weighing the perceived benefits throughout the consumption process against the costs paid when obtaining products or services (48). Value is a subjective construct, and different people have different perceptions of value. For example, the older adults with chronic diseases will think that the most valuable part of remote health management is chronic disease management, but for the older adults without chronic diseases, daily health monitoring is the most valuable. Several studies in the retail field have demonstrated that the perceived value of users has a positive impact on behavioral intention (49, 50). This paper mainly studies the acceptance of health management systems by the older adults under telemedicine. The older adults are unfamiliar with telemedicine management, and if the older adults predict that the system can meet their needs for health management, it will increase the desire to use the system. Therefore, the hypothesis is put forward:

H8: Perceived value has a positive impact on the behavioral intention of the older adults to use remote health management.

#### 3.2.8. Perceived interactivity

Perceived interactivity is defined as “the extent to which users perceive their experience as a simulation of interpersonal interaction and sense they are in the presence of a social other (51).” McMillan envisioned perceived interactivity as three overlapped dimensions: two-way communication, control of navigation/choices, and time to load/time to find (52). Since this paper explores the influence factors of the older adults' acceptance of remote health management, it is necessary to combine the characteristics that remote health management is a new concept in China, and define the perceived interactivity in this paper as “the information exchange experience that the older adults expect to perceive in the face of remote health management system.” Jee and Lee (53) found that there is a positive relationship between perceived interactivity and attitudes toward the website. The older adults often need a straightforward and efficient experience due to physiological and psychological reasons. When they think that the information exchange method of the system is in line with their usage habits, they will be more willing to use the system. Therefore, the hypothesis is put forward:

H9: Perceived interactivity has a positive impact on the behavioral intention of the older adults to use remote health management.
H10: Perceived interactivity has a positive impact on the use behavior of the older adults to use remote health management.

#### 3.2.9. Individual innovation

Individual innovation is the ability of an individual to be good at discovering and accepting new things. It is used to assess an individual's acceptance of new things (54). Individual innovation is an important indicator to measure the acceptance of new products or technologies by users. AI Busaidi found that in the context of e-learning, learners with more innovative spirit had a positive perception and usefulness of e-learning (50). This paper mainly studies the acceptance of health management systems by the older adults under telemedicine. Therefore, the older adults with strong individual innovation are more sensitive to new information technology and more easily accept and adapt to the integration of telemedicine and health management. Such older adults often pay more attention to the development of new technologies and can give feedback on the current development trend or service experience of telemedicine, and their risk perception ability is stronger than that of others. The older adults with strong individual innovation have relatively high operational ability, their interactive experience perception of the new system is also more real, and the tendency of use is higher. Therefore, the hypothesis is put forward:

H11: Individual innovation of patients positively impacts effort expectation of behavioral intention to use remote health management services.
H12: Individual innovation of patients positively impacts perceived risk of behavioral intention to use remote health management services.
H13: Individual innovation of patients positively impacts perceived value of behavioral intention to use remote health management services.
H14: Individual innovation of patients positively impacts perceived risk of behavioral interactivity to use remote health management services.

## 4. Materials and methods

### 4.1. Research objects and questionnaire

According to the preset model diagram (Figure 1) and the hypothesis analysis of satisfaction, the study questionnaire of hospital use for patients is divided into two parts. The first part is to collect the basic information and characteristic information of users, including gender, age, educational background, income, frequency of visits, and previous use. The second part is measurement items (Table 1) which are scored on a Likert 5-level scale from 1 to 5 (from strongly disagree to strongly agree). The study was conducted in a level-A tertiary hospital in Shanghai. Before the formal distribution of the questionnaire, on-site investigation and interview were conducted in the hospital to correct the unclear questions, and finally a large number of questionnaires were distributed to the target group. Inclusion criteria: (1) patients aged ≥ 60 years old; (2) patients who are able to answer the questionnaire independently; (3) patients who provide informed consent and are willing to participate in this study. Exclusion criteria: (1) patients with doctor-patient disputes; (2) critically ill emergency patients. A total of 402 valid questionnaires were collected, including 249 males (61.9%) and 153 females (38.1%) with 73 years median age (IQR: 72–79). Among the respondents, 106 (26.4%) had education below junior high school, 117 (29.1%) had high school education, and 179 (44.5%) had were college education or above; 194 (48.3%) lived with their spouses, 142(35.3%) lived with their families, and 66 (16.4%) subjects lived alone.

**Table 1:** Measurement items of patient satisfaction research questionnaire.

**Construct**	**Item**	**Content**
Performance expectation	PE1	RHM is helpful for me to judge my health status.
	PE2	The use of RHM can improve the efficiency of my daily health management.
	PE3	The use of RHM can improve the efficiency of medical service visits.
	PE4	The use of RHM services can improve my health level and quality of life.
Effort expectation	EE1	I am easy to accept RHM services.
	EE2	Learning to use RHM is easy for me.
	EE3	The telemedicine services I have been contacted with are easy to operate.
Social influence	SI1	Media publicity will promote my use of RHM.
	SI2	The use and recommendation of people around me (family members, friends and children) will promote my use of RHM.
	SI3	Hospitals, communities and policy implementation will promote my use of RHM.
Facilitating conditions	FC1	I have basic access devices such as smartphones or tablets, and the RHM platform is compatible with my existing devices.
	FC2	I have sufficient knowledge and experience in using Internet.
	FC3	If I encounter problems when using the RHM platform, I can get the necessary support and help.
	FC4	The use of RHM platform is very consistent with my health management method.
Individual innovation	II1	I am always curious about new things.
	II2	I will take the initiative to focus on new information products and technologies.
	II3	I think it is very interesting to try new and emerging products.
	II4	In my circle of friends, I am usually the first to try out new Internet peripherals.
Behavioral intention	BI1	Even if I have no health problems at present, I am willing to use health management services for monitoring and prevention.
	BI2	When I have health problems, I am willing to use RHM service for health management.
	BI3	I would like to recommend RHM service to others.
Usage behavior	UB1	I use RHM for remote rehabilitation.
	UB2	I use RHM for chronic disease management.
	UB3	I use RHM for health monitoring.
	UB4	I use RHM for accidental injury prevention.
Perceived risk	PR1	The use of RHM platform will cause property damage to me.
	PR2	It will take more time to use remote health management platform.
	PR3	I worry that the use of RHM platform will put psychological burden on me.
	PR4	The use of RHM platform will reveal my personal information, privacy and health data.
	PR5	I am worried that the RHM system is not fully functional, which will affect my health.
Perceived value	PV1	The health monitoring services in RHM are valuable to me.
	PV2	The use and recommendation of people around me (family members, friends and children) will promote my use of RHM.
	PV3	The use of RHM platform will put psychological burden on me.
	PV4	I think the accidental injury prevention services in RHM are valuable to me.
Perceived interactivity	PI1	I hope the doctor-patient communication path of the RHM platform is smooth.
	PI2	I hope to interact with other older individuals on the RHM platform.
	PI3	I hope that the structural framework of the RHM platform is reasonably distributed and easy to operate.
	PI4	I hope the visual performance of the RHM platform is clear.

PE, Performance expectation; EE, Effort expectation; SI, Social influence; FC, Facilitating conditions; II, Individual innovation; BI, Behavioral intention; UB, Usage behavior; PR, Perceived risk; PV, Perceived value; PI, Perceived interactivity.

RHM, remote health management.

### 4.2. Data analysis

The internal consistency of the construct was assessed by Cronbach's α reliability (acceptable if >0.7) and structural reliability (acceptable if >0.6) (55, 56). The convergence and discriminant validity of the measurement model were evaluated by confirmatory factor analysis. If all terms' factor loads were significant and >0.50, the convergence effectiveness was verified (57). Structural equation models were used to test hypothetical models. The eight most widely used fitting indexes in SSCI studies were used to evaluate the overall model fit. The ratio between chi-square statistics and the degree of freedom (χ^2^/df <3), the χ^2^ chi-square, the approximate root means square error (RMSEA), the normed fit index (NFI), the Tucker–Lewis index (TLI), the comparative fit index (CFI), the goodness of fit index (GFI), and the adjusted goodness of fit index (AGFI) were compared. The evaluation and analysis of the SEM in the present study was completed with AMOS24.0 (IBM Corporation Armonk, NY, USA) and SPSS 26.0 (IBM Corporation Armonk, NY, USA).

## 5. Results

### 5.1. Analysis of reliability and validity of data

In this study, the factor loading was between 0.61 and 0.98, showing that each question item had reliability; the overall Cronbach's Alpha coefficients in the study were >0.7, indicating high reliability of the data. The composite reliability (CR) ranged from 0.59 to 0.91, which showed good internal consistency for each construct; the average variance extraction (AVE) ranged from 0.51 to 0.85 (as shown in Table 2), which met the criteria, and therefore, all 10 dimensions had good reliability and convergent validity.

**Table 2:** Reliability and validity analysis of the measurement model in this research.

	**Item**	**Factor load**	**Cronbach's α**	**CR**	**AVE**
II	II1	0.944	0.791	0.596	0.852
	II2	0.655			
	II3	0.770			
	II4	0.685			
EE	EE1	0.937	0.772	0.827	0.620
	EE2	0.721			
	EE3	0.680			
PE	PE1	0.984	0.854	0.919	0.740
	PE2	0.817			
	PE3	0.795			
	PE4	0.832			
SI	SI1	0.769	0.788	0.874	0.699
	SI2	0.798			
	SI3	0.932			
BI	BI1	0.931	0.801	0.896	0.743
	BI2	0.732			
	BI3	0.862			
UB	UB1	0.775	0.702	0.806	0.511
	UB2	0.612			
	UB3	0.719			
	UB4	0.744			
FC	FC1	0.782	0.734	0.898	0.787
	FC2	0.826			
	FC3	0.799			
	FC4	0.904			
PR	PR1	0.974	0.843	0.911	0.721
	PR2	0.816			
	PR3	0.761			
	PR4	0.831			
PV	PV1	0.742	0.788	0.862	0.612
	PV2	0.738			
	PV3	0.709			
	PV4	0.923			
PI	PI1	0.645	0.746	0.847	0.591
	PI2	0.677			
	PI3	0.878			
	PI4	0.931			

PE, Performance expectation; EE, Effort expectation; SI, Social influence; FC, Facilitating conditions; II, Individual innovation; BI, Behavioral intention; UB, Usage behavior; PR, Perceived risk; PV, Perceived value; PI, Perceived interactivity.

In this study, the rigorous AVE method was used to test the discriminant validity of the measurement model. If the square root of AVE for each construct is larger than the correlation coefficient between the constructs, it indicates that the model has discriminant validity. As shown in Table 3, the root mean square of AVE for diagonal constructs in this study is larger than the correlation coefficient outside the diagonal line, so the majority of constructs in this study have good discriminant validity.

**Table 3:** Discriminant validity of the measurement model.

	**AVE**	**FC**	**SI**	**II**	**EE**	**PI**	**PV**	**PR**	**PE**	**BI**	**UB**
FC	0.687	0.829									
SI	0.699	0.304	0.836								
II	0.852	0.151	0.129	0.923							
EE	0.620	0.048	0.041	0.321	0.787						
PI	0.591	0.027	0.023	0.182	0.058	0.769					
PV	0.612	0.058	0.049	0.381	0.122	0.069	0.782				
PR	0.721	−0.014	−0.012	−0.093	−0.030	−0.017	−0.036	0.849			
PE	0.740	0.020	0.017	0.132	0.411	0.024	0.050	−0.012	0.860		
BI	0.743	0.045	0.080	0.193	0.067	1.058	0.053	−0.018	0.046	0.862	
UB	0.511	0.739	0.261	0.223	0.074	0.655	0.074	−0.021	0.041	0.640	0.715

PE, Performance expectation; EE, Effort expectation; SI, Social influence; FC, Facilitating conditions; II, Individual innovation; BI, Behavioral intention; UB, Usage behavior; PR, Perceived risk; PV, Perceived value; PI, Perceived interactivity.

### 5.2. Structural model fit

The various fit indexes and allowable ranges as well as the model fit were as follows: Normed Chi-sqr (χ^2^/df) had an allowable range of 1 <χ^2^/df <3 and a model fit of 2.78; RMSEA had an allowable range of <0.08 and a model fit of 0.07; NFI had an allowable range of > 0.8 and a model fit of 0.89; TLI had an allowable range of > 0.8 and a model fit of 0.92; CFI had an allowable range of > 0.8 and a model fit of 0.84; GFI had an allowable range of > 0.9 and a model fit of 0.94; and AGFI had an allowable range of > 0.8 and a model fit of 0.92. According to the allowable ranges, each degree of fitting of this model meets the requirement.

### 5.3. Validation of structural equation model hypothesis

**Figure 2:**
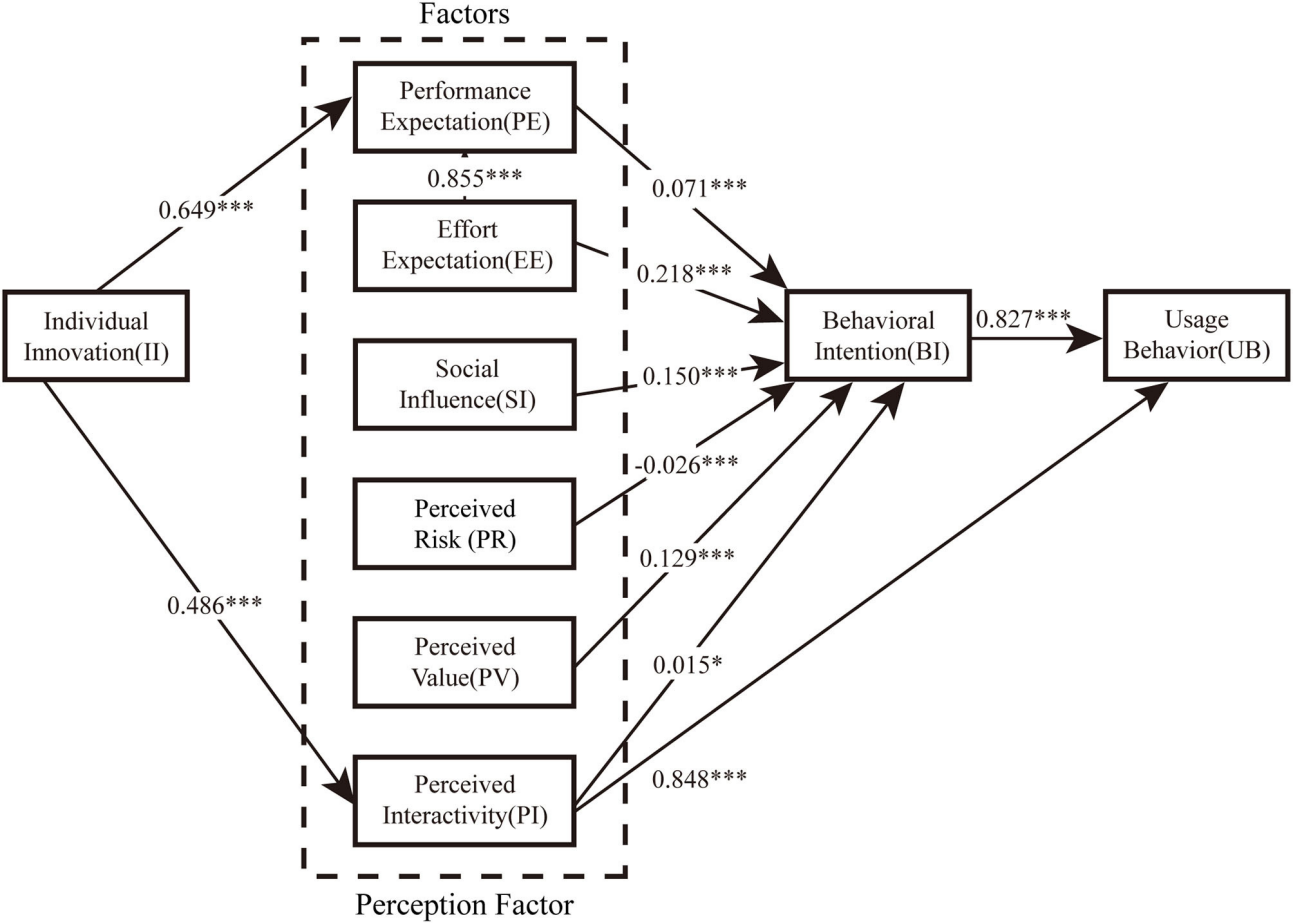
The final model with parameter estimates of the significant paths. *p < 0.05 and ***p < 0.001.

Figure 2 shows the results of the estimated structural model and Table 4 summarizes the results of the hypothesis test. The results of the statistical analysis in this study basically supported the model hypothesis. From the structural path of Figure 2 and the hypothesis test of Table 4, it can be seen that individual innovation has no effect on perceived value and perceived risk, that is, H12 and H13 are invalid. H5 is not supported and facilitating conditions have no effect on applicable behavior. Except this, all other 11 hypotheses are supported. The standardized coefficient indicates that the influencing factors of the behavioral intention of the older adults to accept the health management system are: effort expectation (0.218), social influences (0.150), perceived value (0.129), performance expectation (0.071), perceived interactivity (0.015) and perceived risk (-0.026). At the same time, effort expectation has a significant positive impact on performance expectation. Individual innovation positively impacts performance expectation (0.649) with perceived interactivity (0.486). Perceived interactivity (0.848) and behavioral intention (0.827) have a significant positive effect on the use behavior of the older adults, while the facilitating conditions have little effect on the use behavior.

**Table 4:** Path analysis and detection results of the structural model.

**Hypotheses**	**Structure pattern path**	**Estimate**	**SE**	**CR**	***p*-value**	**Supported?**
H1	PE → BI	0.071	0.018	3.867	***	Support
H2	EE → BI	0.218	0.033	6.511	***	Support
H3	EE → PE	0.855	0.225	3.803	***	Support
H4	SI → BI	0.150	0.014	10.661	***	Support
H5	FC → UB	−0.028	0.024	−1.140	0.254	Not support
H6	BI → UB	0.827	0.086	9.628	***	Support
H7	PR → BI	−0.026	0.009	−2.811	0.005	Support
H8	PV → BI	0.129	0.022	5.803	***	Support
H9	PI → BI	0.015	0.008	1.906	0.047	Support
H10	PI → UB	0.848	0.143	5.910	***	Support
H11	II → PE	0.649	0.112	5.769	***	Support
H12	II → PR	−0.179	0.096	−1.857	0.063	Not support
H13	II → PV	0.240	0.063	3.783	0.061	Not support
H14	II → PI	0.486	0.109	4.456	***	Support

PE, Performance expectation; EE, Effort expectation; SI, Social influence; FC, Facilitating conditions; II, Individual innovation; BI, Behavioral intention; UB, Usage behavior; PR, Perceived risk; PV, Perceived value; PI, Perceived interactivity.

## 6. Discussion

### 6.1. Primary findings

With the rapid increase in demand for health/healthcare information and services, it has led to the global promotion of user-oriented and patient-centered healthcare in the era of information technology. In the global aging wave, telemedicine management systems are expected to play an important role in health care services for the older adults. However, to truly achieve this goal, many aspects, from the design of the application to the factors that affect the behavioral intention, should be carefully considered. In this study, the traditional UTAUT model is extended and four variables are added: individual innovation, perceived risk, perceived value, and perceived interactivity, so that it can more comprehensively explain the complex use behavior of specific populations in specific fields.

Performance expectation, effort expectation and social influences all positively impact the behavioral intention of the older adults to use remote health management in this study. This finding is consistent with previous studies (29, 58). The use of the health management system by the older adults is largely determined by the usefulness of the system itself. In China, traditional medical concepts are deeply rooted in the older adults and also affect the mode of medical treatment. In the system design, let the older individuals perceive that the health management system can improve their own health management efficiency, meet their own actual needs and is beneficial to their own health and is easily to use, the more motivated they are to use it. On the other hand, the perceived usefulness depends on the perceived ease of use, that is, the simpler the technology to use, the more useful it is (59). Telemedicine institutions should pay attention to the cultivation of skills and habits of the older individuals, optimize the process of telemedicine services, improve the perceived ease of use of older individuals, and meet the expectations of the older adults. The older adults live in a social environment, and the impact of society on the older adults cannot be ignored. The interaction between the older adults and their communication groups is one of the factors to encourage the use of health management, and can alleviate the resistance to new technologies brought about by technical pressure (60). When people around them (such as friends, family members and colleagues) mention or use the new system more, the older individuals will be influenced by them and subjectively increase their acceptance of the new system, resulting in their willingness to try, and their behavioral intention to use it will increase.

Perceived value and perceived interactivity positively affect the behavioral intention of the older adults to use remote health management, while perceived risk negatively impacts their behavioral intention to use remote health management. All of these variables reflect the intuitive perception of new technologies in the older individuals. In general, the needs of different older adults for health management are different. The perceived value transmitted by remote health management to the older individuals has a positive impact on the development of remote health management. In order to improve the perceived value, it is necessary to give priority to provide higher value to the older adults and guide the older adults to develop habits. The older adults are relatively sensitive to risk perception. In addition to the security issues related to mobile payment, remote health management also stores a large amount of personal information of the older adults. Some older individuals will be skeptical about the information quality content in the remote health management system, or worry about misdiagnosis or diagnosis and treatment errors in online consultation, which will become hidden dangers affecting the promotion and use of remote health management. The older adults are more inclined to seek objective and verified sources of information, a feature that is more pronounced in technology-related activities. The use habits of the older adults in China are cautious and conservative. The remote health management system is a relatively new thing for them, they have a low trust in new things, and believe that they will experience errors and losses during use. Therefore, for the problems worried by the older adults, measures such as strengthening the construction of health management system and improving laws and regulations can ensure the basic rights and interests of the older adults, reduce their perception of risks, and improve their behavioral intention to use.

Perceived interactivity has previously been less analyzed in the context of telemedicine. In this study, it plays an important role in both behavioral intention and use behavior. Therefore, it is necessary to reflect characteristic services in functional design and interface design, pay attention to providing sufficient information tips for the older adults on the Interaction Point. The design should be conducted from perceived usefulness, perceived safety, perceived ease of use and perceived pleasure, so that users have strong curiosity and strong interest in remote health management service systems and user loyalty may be improved. It is worth noting that innovation in the older adults also affects the use behavior through perceived interactivity. Some older individuals with high acceptance of new things often have higher expectation for the applicability of emerging technologies and pay more attention to the innovation and development of technologies. They are usually eager to stay ahead of others and become the earliest adopters of new products with higher behavioral intention to use the health management system. Therefore, in the process of system popularization and use, we should give full play to the leading role of the older adults with strong individual innovation, and further strengthen the influence of social influences and facilitating conditions.

Unlike previous studies, facilitating conditions in this study do not influence use behavior. The reason for this is that in the current intelligent social situation, the use of smartphones by the older adults is increasing, with the resources and conditions for the use of health management systems; secondly, the population using the health management system is the older adults. The system design for the older adults is relatively simple and easy to operate, as well as the health management system is equipped with operating video and consultation services. In case of difficulties, the older adults can get help. With conditions and resources available, the older adults believe that the conditional factors no longer affect the use of the health management system.

### 6.2. Value of models in the design of Internet healthcare systems

Based on the technology integration model, perceived interactivity has a significant impact on behavioral intention to use and use behavior, which provides inspiration for optimizing remote health management design and implementation. It should consider the important role of interactive design and user experience in the process of product design of the remote health management system while using related technologies. The design of remote health management mainly starts from the following aspects: firstly, it should perform user analysis for the older individuals with strong individual innovation and high effort expectation to determine their interest preferences and attitude toward the development of telemedicine trends; secondly, it should conduct functional positioning based on performance expectation, and performance positioning of facilitating conditions based on perceived value; thirdly, in the design stage, it should comprehensively consider whether the design system process operation is simple, convenient and efficient based on perceived interactivity, and consider safety issues such as privacy protection, functional perfection, and diagnostic professionalism based on perceived risk.

By integrating UTAUT model and remote health management system design, the influencing factors of behavioral intention to use and subsequent design scheme can be considered in all directions and multiple levels. This model can guide researchers to tap the needs and preferences of older adults groups, so as to accurately grasp user needs, enhance user viscosity, improve service experience, and improve the value of remote health management system. For the functional positioning of remote health management systems, it requires to comprehensively consider task-orientation and social-orientation, simplify the process of using medical services and improve the efficiency of users' visits.

#### 6.2.1. Optimizing the system design from the perspective of interactive design

The interaction process runs through the whole remote health management service. Doctors and patients achieve effective diagnosis and treatment results through the cooperation of software and hardware interaction system. It should optimize the telemedicine service process, so that patients can conveniently obtain telemedicine services, improve the perceived ease of use, and meet the efforts expectation. The design needs to be patient-oriented, from the perspective of “matching” to carry out the design and development of service equipment and systems. It should pay attention to the convenience and interface friendliness of telemedicine terminal service system operation for the adults, and continuously improve the functions and types of telemedicine services.

This study shows that the behavioral intention of the adults to use the remote health management system is largely affected by the effort expectation. In other words, ease of use has a more important impact on whether the adults use the remote health management system. With the increase of age, the adults have different psychological and physiological characteristics from other age groups. The user interface is the medium through which users and machines exchange information with each other, including the input and output of information. On the basis that the user interface and interaction conforming to the physiological characteristics of the adults, designers should also pay attention to the psychological feelings of the older adults during the use process, including cognitive load and user experience. Cognitive load refers to the working memory requirements caused by complex tasks in specific situations that require novel information or novel processing methods (61). It is an important indicator of cognitive feedback in the interactive process. In this paper, it refers to the cognitive processing degree of the adults using remote health management operations. In the design, it is necessary to enhance the acceptance of remote health management by the adults, reduce the cognitive load (62) by using the navigation structure of linear hierarchical navigation (63), and match the operation logic of remote health management with the interactive experience of the inherent linear visual exploration mode of the adults.

Physiologically, due to the decline in tactile sensitivity and muscle control, it will be very difficult for the older adults to complete certain fine movements such as button operations. At the same time, due to visual nerve degeneration and reduced pyramidal cells of the retina, the color identification ability of the adults becomes worse. Psychologically, the older adults believe that the remote health management system is a high-tech product and requires a new knowledge system to control, so there is a widespread technological gap in science and technology. In view of these characteristics, the steps in the use process should be simplified as much as possible to reduce the cognitive burden of older users, so that they can easily use the system; in the necessary information layered design, different colors are used to distinguish functional modules, and color systems with medium brightness and high brightness contrast are used; at the same time, for the fluency of the product, it needs to comprehensively consider finger operation, user thinking and page conversion to ensure smooth connection during operation; the combination of simple one-finger gestures (such as click and drag) with compatible user interface elements (such as buttons and scales) is more helpful for the learning, memory and execution of the system (64). The older individuals have significantly higher subjective preferences for specific type icons (i.e., skeuomorphic) than abstract type icons (i.e., flattened) (65), so when patients input information, it should minimize text input and improve voice input functions; when patients are searching for conditions for chronic disease management, it should provide relevant treatment experience sharing and relevant physical icons and data of the disease. At the same time, emergency medical consultation options or shortcuts should be set up, such as shortcut keys to alarm for help, voice for help, etc., to meet the consultation and rescue needs of some accidental injuries and sudden disease conditions. Meanwhile, the graphic layout of remote health management should respect the flow of sight in the Chinese reading context, especially when designing timelines and other graphic representations with flow direction, it should respect the reading habits of the older adults.

**Figure 3:**
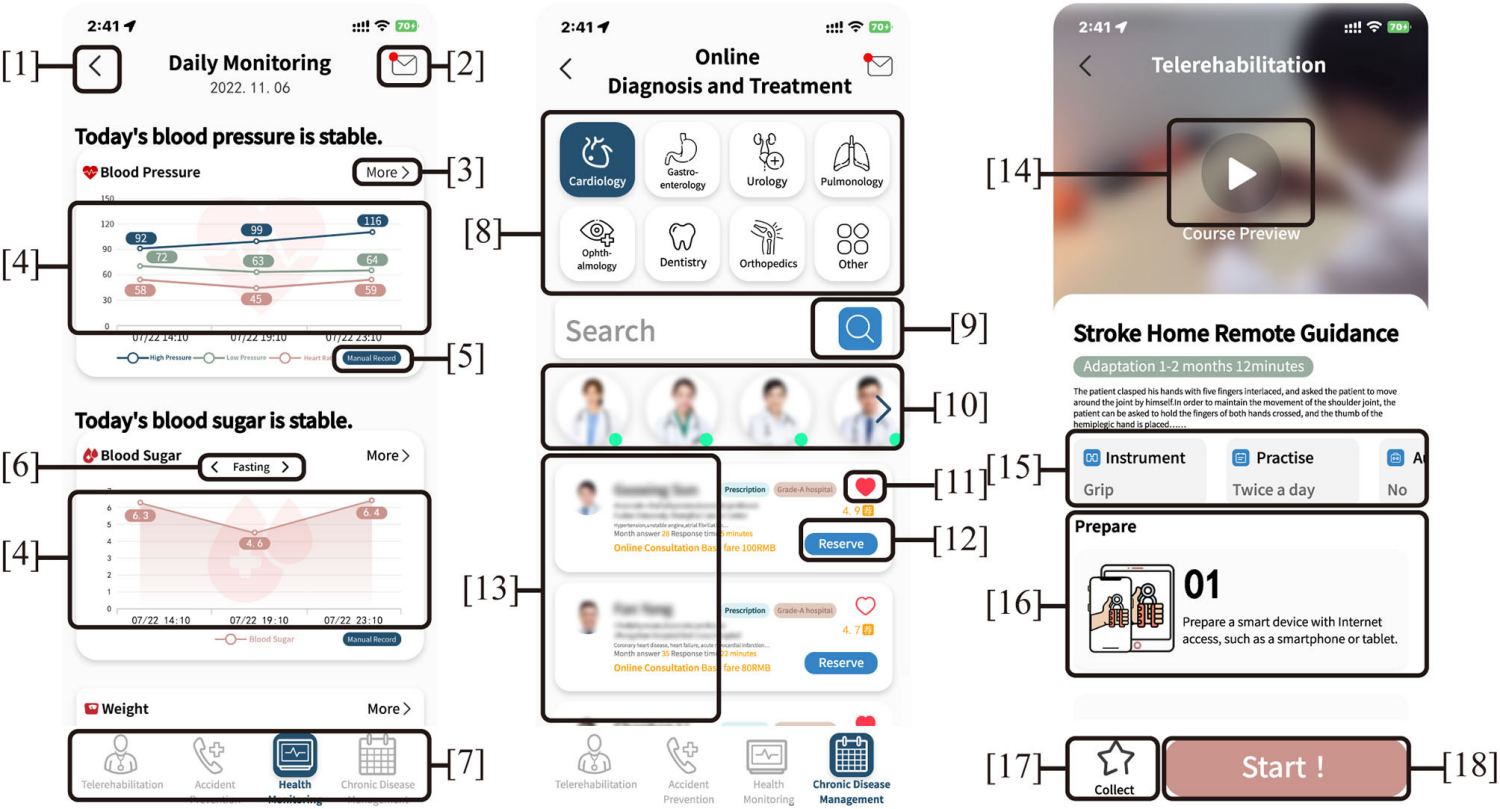
Design patterns of Interface: (1–3, 5, 6, 9, 11, 12, 14, 17, 18) assisted navigation buttons, (4) management interface, (7) tab menu, (8) springboard, (10) gallery, (13) lists, (15,16) cards.

In order to meet the performance expectation, the functional modules mainly come from the requirements of hospitals and social needs, not only including the four core functions used by the older adults: health monitoring, chronic disease management, rehabilitation management, accidental injury prevention, but also considering the user's use habits, user experience, behavioral characteristics and visual effects in order to meet the multi-level needs of remote health management system such as usefulness, ease of use, availability, and convenience. Designers need to solve specific problems in user interface design to lower the cognitive threshold for initial use by the older users, enhance the visibility of current functions in the interface, and provide adequate support to users. The visualization and ease of use of various physiological data have an important impact on the reception and transmission of information by both doctors and patients. The operability and comfort of the hardware system are also the basis for efficient communication between both sides. System development designers can organize user interfaces in a meaningful and useful way by putting relevant content together and separating irrelevant content, and dividing different content with different colors and fonts. These optimized hierarchical interfaces can enable users to browse content and use functions in a simpler way. In terms of navigation, the user memory load should be reduced through information visualization and simplified user interface and following a hierarchical structure of step-by-step into details, especially those tasks that require more steps. Designers should clearly display the task status and name of the current interface, and let the user know the progress of the task to enhance the user's control (see Figure 3).

In conclusion, in health management services, it is necessary for patients to feel that the remote health management service systems can optimize the use process and improve the treatment experience, and perceive the value and convenience of the product service experience. Secondly, for the designed management system, some older individuals can be invited to use it, and then continue to explore, improve, innovate, and design a system that satisfies the older adults according to the feedback results of the older adults after use, and continuously improve the emotional design level of the operating system.

#### 6.2.2. Apply the service design concept to remote health management services

**Figure 4:**
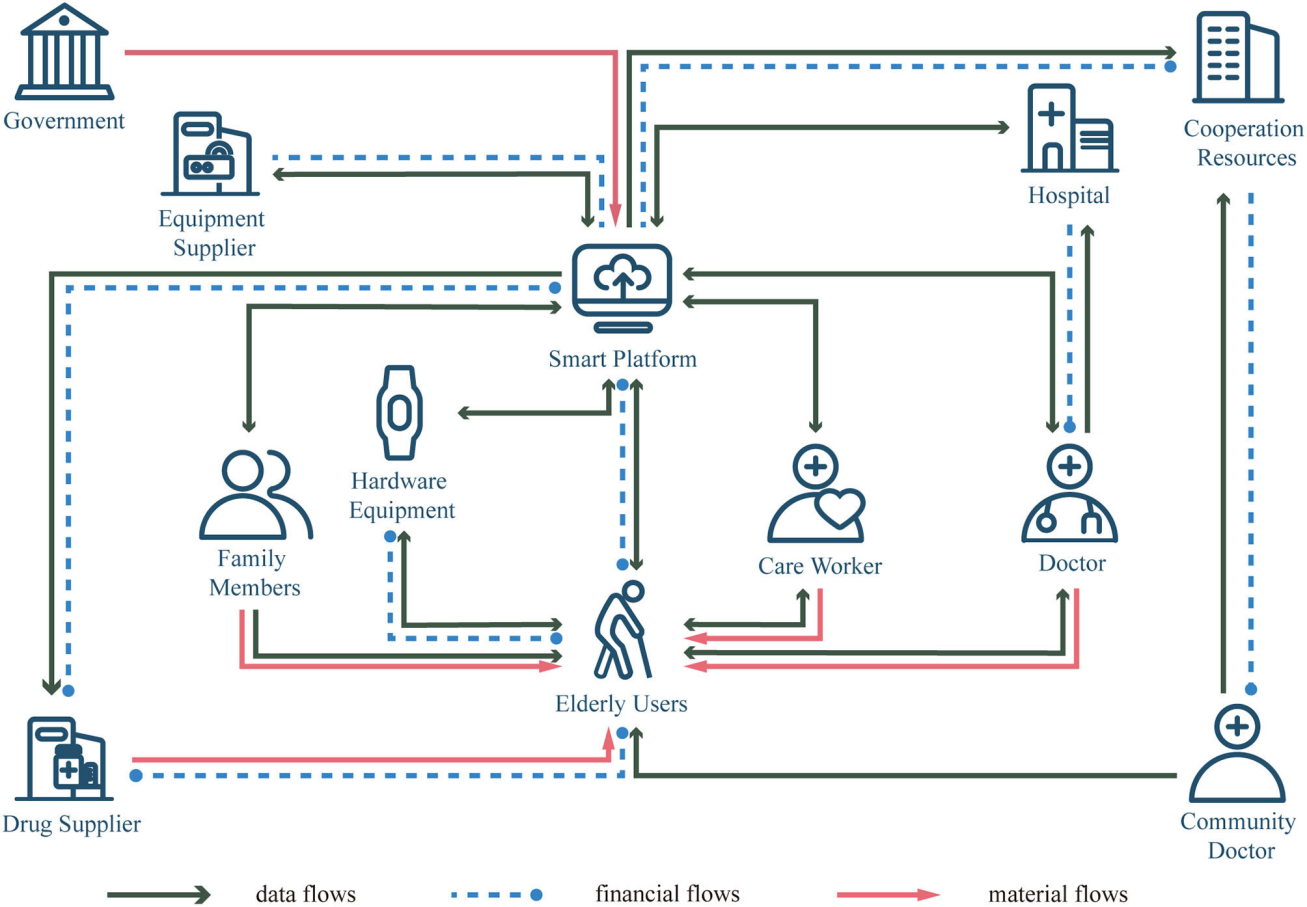
The whole interaction process of the telemedicine-based older adults remote health management service system.

Health management is a professional and complex medical service, which involves the systematic association and dynamic management of individuals, families, communities and hospitals. The older adults encounter many problems in the process of health management, and single products or services often fail to meet the diverse needs of older individuals and cannot ensure the continuity of service experience. Service design, as a social frontier system design theory for solving problems systematically, is a design phenomenon that includes tangible and intangible conditions and multiple interactions. It is necessary to integrate tangible products and intangible services into a complete service system to provide effective working ideas for the urban aging health management system. In the service system of older health management, the older adults are both service recipients and service providers, and they can realize their own value while receiving services. Due to the complexity of health management content, in order to meet the multi-dimensional needs of users for chronic disease health management, it is necessary to carry out whole-process design for health management, create service modules with different contents, build a closed-loop of whole-process service experience for sustainable development and all-round management, and provide the older adults users with a whole-life cycle health management program (Figure 4). In the older health management service system, it should mobilize the enthusiasm of all stakeholders and stimulate creativity through various operations, enhance the viscosity between the system and users, and help all stakeholders establish a lasting and reliable relationship, so as to ensure the sustainable development of the service system, build a closed-loop of whole-process services for sustainable development, and establish a telemedicine-based older remote health management service system.

## 7. Limitations

Based on the UTAUT model, this study constructs the influence factor model of the acceptance of the health management system for the older adults, and through questionnaire and data analysis, some research conclusions are obtained, but there are still some shortcomings.

Firstly, this study only investigated the older adults attending a level-A tertiary hospital in Shanghai. in Shanghai. Although this group is representative, due to geographical limitations and research resources, fewer users in other provinces and cities are surveyed. In order to ensure that the findings are more general and generalizable, sample size should be expanded in future studies. At the same time, other countries or regions can also be considered to deeply explore the development mode of telemedicine in each country, so as to provide reference for the development of telemedicine in China, and gradually improve the unequal state of medical resources in various regions of China.

Secondly, this study uses a cross-sectional study to identify factors that affect the continuous acceptance of health management systems by the older adults, and later studies can use longitudinal studies to explore the degree of acceptance of the health management system by the older adults over time.

## 8. Conclusion

With the help of UTAUT model, this paper constructs the behavioral intention model of remote health management for the older adults, makes the research logic clearer and more reasonable, and excavates the new function and perspective of UTAUT model. This research model proves that effort expectation, performance expectation, perceived risk, perceived value and perceived interaction are the important factors that affect the older adults' use of remote health management services. Our findings are conducive to the online health management platform's selection of appropriate business methods and priorities, improvement of the service content and construction, so as to improve the medical experience and promote the use intention of older adult users. At the same time, it provides theoretical support for building a good Internet-based medical platform, which will promote better use of remote health management platform under in the future.

Based on the research results, this paper provides reference and basis for better use of remote health management services by the older adults under the internet environment, which has practical significance for optimizing the uneven distribution of resources in China's medical and health fields. In addition, this research also provides important insights for understanding the acceptance behavior of older adult users in the field of telemedicine and how to improve the older individuals experience from a design perspective.

## Data availability statement

The raw data supporting the conclusions of this article will be made available by the authors, without undue reservation.

## Ethics statement

The studies involving human participants were reviewed and approved by the Ethics Committee of Shanghai East Hospital. The patients/participants provided their written informed consent to participate in this study.

## Author contributions

WL and JG designed the study question, performed the statistical analyses, and wrote the original version of the manuscript. WL, XL, TZ, and JY performed the investigation. WL, JY, and JG designed the questionnaire and tables. QT was responsible for the overall supervision of the study and the revision of the manuscript. All authors read and approved the final manuscript.
